# Supplementary material for: An Extracellular/Membrane-Bound S100P Pool Regulates Motility and Invasion of Human Extravillous Trophoblast Lines and Primary Cells
Source: Biomolecules. 2023 Aug 9;13(8):1231. doi: 10.3390/biom13081231 (PMC10452538; doi:10.3390/biom13081231)

Supplementary Figure S1

Figure 3A

Jeg-3 cells

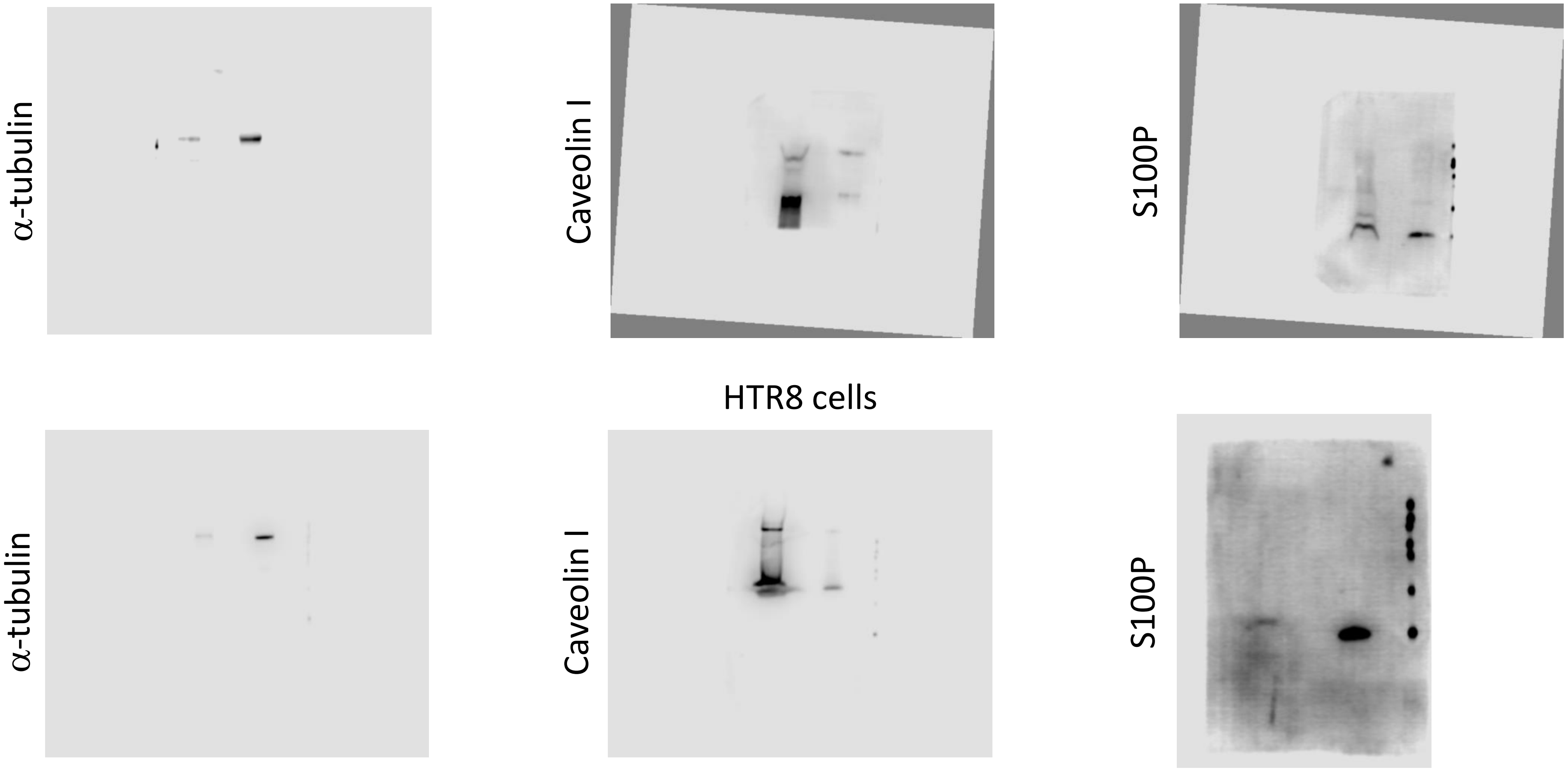

HTR8 cells

Figure 3A

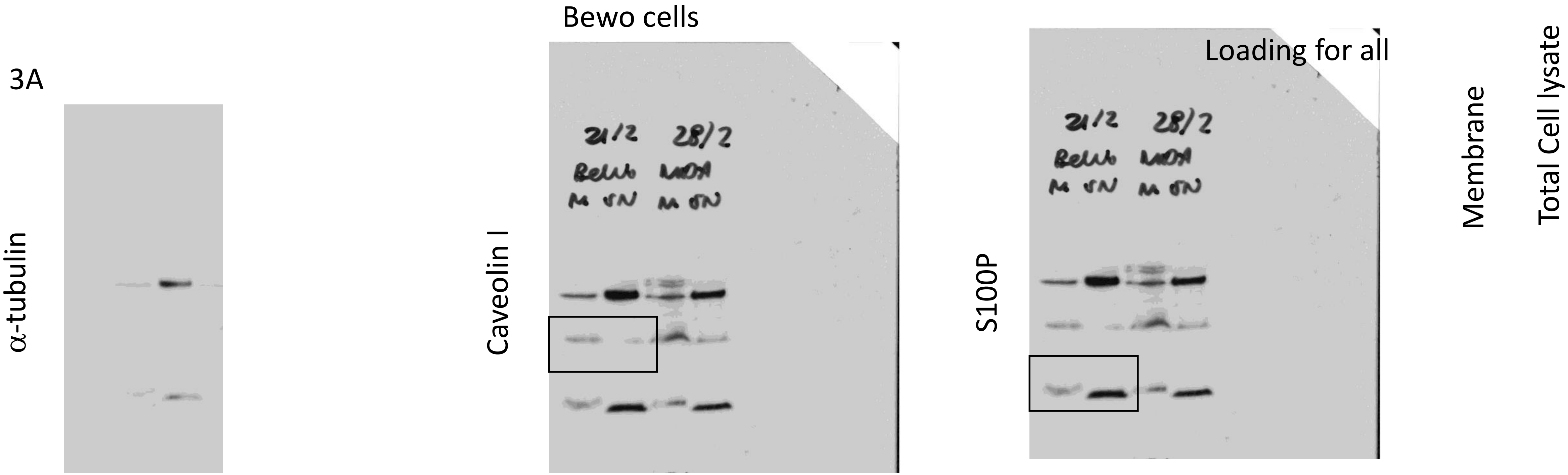
Bewo cells
Loading for all
α-tubulin
Caveolin I
S100P
Membrane
Total Cell lysate
21/2
28/2
BeWo
MDA
M SN
M SN

Figure 3B

S100P biotinylation

Loading for all

Total Cell lysate

Biotinylated Membrane

Jeg-3 cells

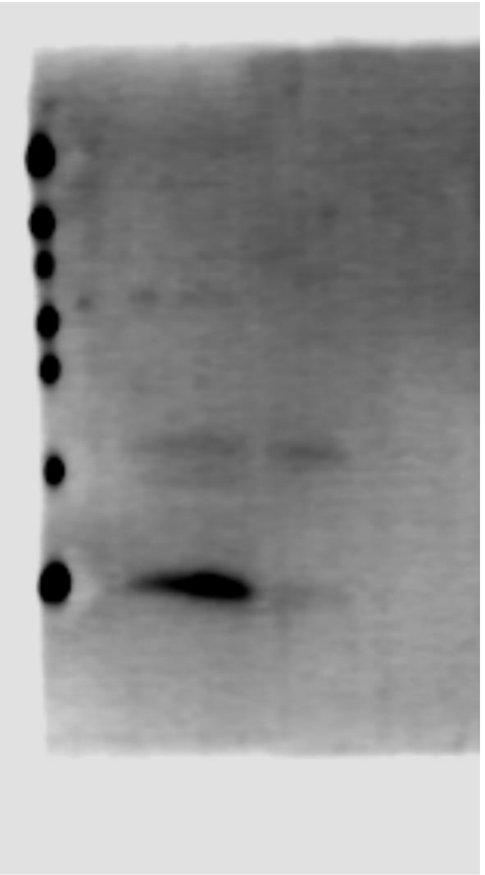

Bewo cells

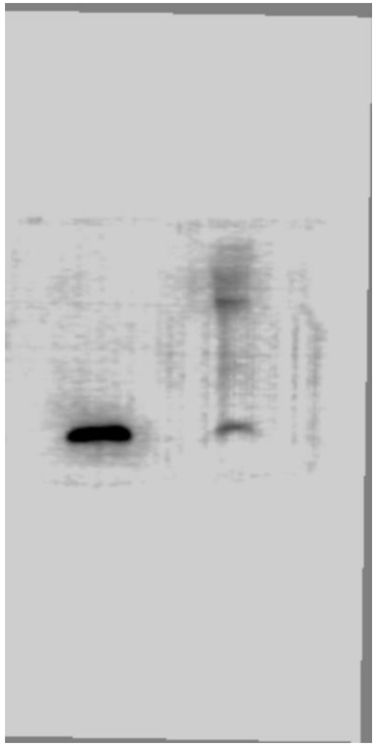

HTR8 Clone 7 cells

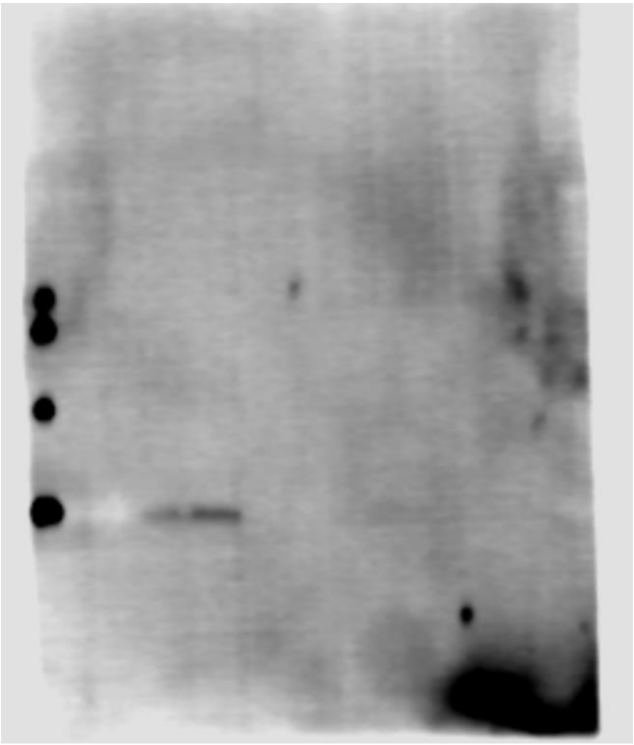

Figure 3C

S100P biotinylation

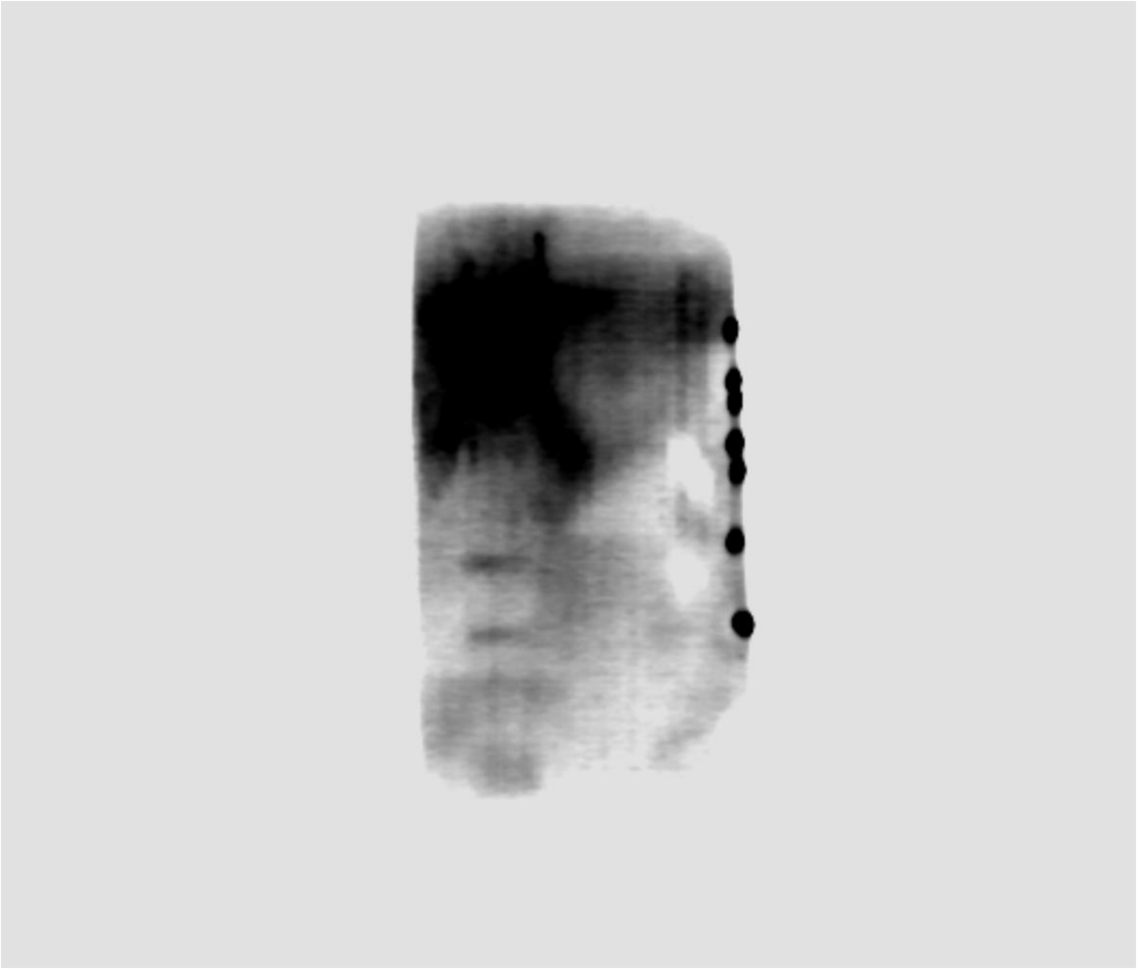

Supplement: Supplementary file 1 [file biomolecules-13-01231-s001.zip › biomolecules-2407713 figure S1.pdf]
