# Supplementary material for: An Extracellular/Membrane-Bound S100P Pool Regulates Motility and Invasion of Human Extravillous Trophoblast Lines and Primary Cells
Source: Biomolecules. 2023 Aug 9;13(8):1231. doi: 10.3390/biom13081231 (PMC10452538; doi:10.3390/biom13081231)
Supplement: Supplementary file 1 [file biomolecules-13-01231-s001.zip › biomolecules-2407713-TableS S1 and S2.pdf]

Supplementary Table S1: Antibodies used in this study.

Antibody	Experiment	Type	Clone	Supplier	Dilution	Incubation	Buffer
HLA-G	IHC	Monoclonal	IgG1 Mouse	Abcam	1/100	Overnight at 4^{0}C	2.5% goat serum
S100P	IHC	Monoclonal	IgG Rabbit	Abcam	1/1500	Overnight at 4^{0}C	2.5% goat serum
S100P	IF	Monoclonal	IgG Mouse	BD Bioscience	1/200	45 minutes at room temperature or 60 minutes at 37^{0}C	1% Goat serum
β1 integrin	IF	Monoclonal	IgG1 Mouse	Millipore	1/200	45 minutes at room temperature	1% Goat serum
Paxillin	IF	Monoclonal	Mouse	Invitrogen	1/200	45 minutes at room temperature	1% Goat serum
α-Tubulin	WB	Monoclonal	Mouse	Sigma	1/5000	Overnight at 4^{0}C	3% BSA
S100P	WB	Polyclonal	Goat	R&D	1/1000	Overnight at 4^{0}C	3% BSA
Caveolin I	WB	Monoclonal	Rabbit	Santa Cruz	1/750	Overnight at 4^{0}C	3% BSA
Mouse	WB	Biotinylated	IgG Horse Anti-Mouse	Thermo-Fisher	As per instructions	As per instructions	As per instructions
Rabbit	WB	Biotinylated	IgG Goat Anti-Rabbit	Thermo-Fisher	As per instructions	As per instructions	As per instructions
Rabbit IgG	**Negative control**			Santa Cruz	1/2000	Overnight at 4^{0}C	2.5% goat serum
Mouse IgG	**Negative control**			Santa Cruz	1/100	Overnight at 4^{0}C	2.5% goat serum
Mouse	IF	Monoclonal-FITC	IgG Rabbit Anti-Mouse	Dako	1/100	45 minutes at room temperature	1% Goat serum
Mouse IgG	WB	Monoclonal-HRP	Rabbit	Sigma	1/5000	2 hours at room temperature	3% BSA
Rabbit IgG	WB	Monoclonal-HRP	Mouse	Sigma	1/5000	2 hours at room temperature	3% BSA

Supplementary Table S2: Total number of live cells counted for motility and invasion of Jeg-3, HTR8/SVneo trophoblast cell lines and primary EVT cells after S100P antibody delivery.

**Cell lines**	**Control**		**Mock treatment**	**S100P siRNA treatment**
Jeg3 motility	1002		576	2443
Jeg3 invasion	896		354	188
HTR8/SVneo clone 3 motility	1910		Not applicable	Not applicable
HTR8/SVneo clone 3 invasion	1507		Not applicable	Not applicable
HTR8/SVneo clone 5 motility	1817		Not applicable	Not applicable
HTR8/SVneo clone 5 invasion	2488		Not applicable	Not applicable
**HTR8/SVneo clone 7 motility**	5568		Not applicable	Not applicable
**HTR8/SVneo clone 7 invasion**	6931	Not applicable	4597	Not applicable
**Primary EVT motility**	1604	1375	1074	Not applicable
**Primary EVT invasion**	2238	1814	1317	Not applicable

Jeg-3, HTR8/SVneo clone 3 control cells and HTR8/SVneo clone 5 and clone 7 expressing S100P as well as primary first trimester EVTs cells were either left untreated, mock treated with goat serum or siRNA controls or incubated with anti S100P antibody or S100P siRNA prior to seeding on Boyden chambers for motility or matrigel-coated Boyden chambers for invasion. Data presented in this Table are the sum of all cells counted on the far side of the chambers at the end of the incubation from three or four independent experiments (Figures 4, 5 and 6).
